# 13693 Racial Disparities in Potentially Avoidable Hospitalizations During the COVID-19 Pandemic

**DOI:** 10.1017/cts.2021.613

**Published:** 2021-03-30

**Authors:** Richard K. Leuchter, Chad Wes A. Villaflores, Keith C. Norris, Andrea Sorensen, Sitaram Vangala, Catherine A. Sarkisian

**Affiliations:** UCLA David Geffen School of Medicine

## Abstract

ABSTRACT IMPACT: These findings identify a new way in which the COVID-19 pandemic exacerbates racial/ethnic health disparities, and will thus direct future research to explore potentially avoidable hospitalizations, as well as direct health policy to improve the value of this specific aspect of care without further widening the disparity. OBJECTIVES/GOALS: Racial and ethnic disparities in potentially avoidable hospitalizations predate COVID-19. In order to identify and address healthcare disparities exacerbated by the pandemic, we examined whether and to what extent the pandemic affected numbers of potentially avoidable hospitalizations by race and ethnicity. METHODS/STUDY POPULATION: This single-center pre-post study of 904 patients at UCLA included all patients admitted to an internal medicine service for an ambulatory care sensitive condition (ACSC) between March-August of 2020 (post) and March-August of 2019 (pre). We measured the change in number of potentially avoidable hospitalizations (defined per the Agency for Healthcare Research and Quality guidelines) stratified by race and ethnicity. We calculated 95% CIs for the number of potentially avoidable hospitalizations using a cluster bootstrap procedure, clustering at the level of patients. We inverted the bootstrap CIs to calculate p-values for overall changes within racial/ethnic groups as well as differential changes between groups. Patients with missing or unspecified racial/ethnic data were excluded (n=1,003; 7.8%). RESULTS/ANTICIPATED RESULTS: Between March 1 and August 31, 2020, 347 out of 4,838 hospitalizations (7.2%) were potentially avoidable, compared to 557 out of 6,248 (8.9%) during the same 6-months of 2019. Reductions in potentially avoidable hospitalizations among Non-Hispanic White (-50.3%; 95% CI, -60.9 - -41.2; p<0.001) and Latinx (-32.3%; 95% CI, -59.8 - -12.2%, p<0.001) patients were statistically significant, whereas reductions among African American (-8.0%; 95% CI, -39.9 - +16.2) and Asian (-16.1%; 95% CI, -75.7 - +20.4) patients were not statistically different from 0%. The relative differences in magnitudes of reduction were only statistically significant between African American and non-Hispanic White patients (-50.3% v. -8.0%; 95% CI as above; p=0.015). DISCUSSION/SIGNIFICANCE OF FINDINGS: Racial disparities in potentially avoidable hospitalizations increased during the COVID-19 pandemic at this large urban health system. Healthcare leaders, researchers, and policy makers should focus on efforts to prevent a post-pandemic resurgence of low-value hospitalizations in ways that do not further widen disparities.

